# Diastolic dysfunction is equally common in pre-diabetes and diabetes and associated with concomitant cardiometabolic risk factors

**DOI:** 10.1136/openhrt-2026-004052

**Published:** 2026-05-20

**Authors:** David Kylhammar, Lina Hult, Peter Blomstrand, Hannes Holm Isholth, Amra Jujic, Martin Magnusson, Carl Johan Östgren, Jan Engvall, Kristofer Hedman

**Affiliations:** 1 Department of Health, Medicine and Caring Sciences and Wallenberg Centre for Molecular Medicine, Linköping University, Linköping, Sweden; 2 Department of Clinical Physiology in Linköping, Region Östergötland, Linköping, Sweden; 3 Deptartment of Health, Medicine and Caring Sciences, Linköping University, Linköping, Sweden; 4 Department of Natural Science and Biomedicine, Jönköping University, Jönköping, Sweden; 5 Department of Clinical Sciences in Malmö, Lund University, Malmö, Sweden; 6 Department of Cardiology, Lund University and Skåne University Hospital, Malmö, Sweden; 7 Department of Clinical Sciences in Malmö and Wallenberg Centre for Molecular Medicine, Lund University, Malmö, Sweden; 8 Department of Health, Medicine and Caring Sciences and Center for Medical Image Science and Visualization, Linköping University, Linköping, Sweden

**Keywords:** DIASTOLIC DYSFUNCTION, EPIDEMIOLOGY, Atherosclerosis, Diabetes Mellitus, Metabolic Syndrome

## Abstract

**Aims:**

To investigate the prevalence of diastolic dysfunction and associated risk factors in pre-diabetes and diabetes in the general population.

**Methods:**

Diastolic function was assessed by transthoracic echocardiography in a cross-sectional sample of 3840 men and women aged 50–64 years from the Swedish CArdioPulmonary bioImage Study. Anthropometry, medical history, blood pressure, biochemistry and coronary atherosclerosis assessed by CT were recorded. Population-specific reference ranges were applied. Diastolic function was compared across glycaemic groups and analysed in relation to risk factors.

**Results:**

Normoglycaemia was present in 82%, pre-diabetes in 12% and diabetes in 6%. Diastolic function was impaired (p<0.001) in pre-diabetes and diabetes, with higher prevalence of abnormal diastolic variables in pre-diabetes (30%) and diabetes (33%) than in normoglycaemia (21%). Among participants with pre-diabetes/diabetes, diastolic dysfunction was associated with hypertension and more severe coronary atherosclerosis (p<0.001). In multivariable analyses, waist circumference (OR 1.034, 95% CI 1.013 to 1.054) and high Coronary Artery Calcium Score (OR 2.897, 95% CI 1.373 to 6.113) were independently associated with diastolic dysfunction, also after exclusion of subjects with ischaemic heart disease. In those without coronary atherosclerosis and hypertension, systolic blood pressure was the only independent risk factor (OR 1.027, 95% CI 1.002 to 1.053).

**Conclusions:**

Diastolic dysfunction was as common in pre-diabetes as in diabetes and was mainly associated with central adiposity, hypertension and coronary atherosclerosis. In individuals without coronary atherosclerosis or hypertension, systolic blood pressure was still the only independent predictor. These findings challenge the concept of a clinically relevant isolated diabetic cardiomyopathy and highlight the importance of early and comprehensive cardiometabolic risk factor control to prevent heart failure with preserved ejection fraction.

**WHAT IS ALREADY KNOWN ON THIS TOPIC**

**WHAT THIS STUDY ADDS**

- We show in a large, well-characterised general population sample that diastolic dysfunction was as frequent in pre-diabetes as in established diabetes and associated mainly with hypertension, central adiposity and coronary atherosclerosis, even in the absence of ischaemic heart disease. In individuals without coronary atherosclerosis and hypertension, systolic blood pressure was still the only factor independently associated with diastolic dysfunction.

**HOW THIS STUDY MIGHT AFFECT RESEARCH, PRACTICE OR POLICY**

- These findings suggest that diastolic dysfunction in dysglycaemia is largely linked to concomitant modifiable cardiometabolic risk factors rather than an isolated diabetic cardiomyopathy. The results emphasise the importance of early risk factor management, including weight control and blood pressure optimisation, to prevent heart failure with preserved ejection fraction.

## Introduction

 Diabetes is an endemic disease with an estimated worldwide prevalence of more than 400 million people.^1^ The disease is associated with a twofold to fourfold increase in the risk of developing heart failure^2^ and approximately half of the diabetes-associated mortality is due to cardiovascular disease.^3^

Heart failure is presently classified as heart failure with reduced ejection fraction (HFrEF, left ventricular ejection fraction (LVEF) ≤40%), heart failure with mildly reduced ejection fraction (LVEF 41–49%) and heart failure with preserved ejection fraction (HFpEF, LVEF ≥50%).^4^ HFpEF is increasingly recognised and its frequency is soon poised to exceed the frequency of HFrEF.^5^ HFpEF is a complex clinical syndrome in which impaired diastolic function and consequent abnormalities in left ventricular filling are considered central pathophysiological mechanisms. Diastolic function is primarily determined by myocardial relaxation and chamber stiffness, which depend both on cardiomyocyte properties and extracellular matrix composition, and are influenced by factors such as afterload and myocardial ischaemia. Accordingly, hypertension and coronary artery disease have been associated with HFpEF, but metabolic conditions such as obesity and diabetes mellitus have also been implicated in the pathogenesis of HFpEF.^6 7^

Central obesity, in particular, has been proposed to impair diastolic function through neurohormonal activation and adipokine imbalance, leading to direct myocardial effects as well as promotion of hypertension.^8^ The concept of a diabetic cardiomyopathy was first introduced in the 1970s, describing left ventricular dysfunction in the absence of coronary artery disease and hypertension.^7^ Subsequent studies have suggested that this condition may result from direct myocardial effects of dysglycaemia,^9^ but the existence of a distinct and clinically relevant diabetic cardiomyopathy remains a subject of ongoing debate.^10^

Diastolic function is appraised by Doppler evaluation of transmitral flow and tissue Doppler velocities of the mitral annulus in combination with left atrial size and pulmonary artery pressure from transthoracic echocardiography (TTE).^11^ Based on previous data from five population-based studies including an echocardiographic evaluation of diastolic function, the pooled prevalence of isolated diastolic dysfunction was calculated to be 35% among subjects with diabetes.^12^ Diastolic dysfunction was, however, not unanimously defined in these studies, the prevalence numbers varied greatly and the strong age-dependency of diastolic function variables was not taken into account. One study has investigated the ‘true’ prevalence of HFpEF (ie, objective signs of diastolic dysfunction together with symptoms of heart failure) among subjects with diabetes in the general population and found it to be 23% in subjects >60 years of age,^13^ a number much higher than that reported for community residents in general.^14^ Epidemiological data on diastolic function in pre-diabetes is rare.^15^,^17^

By using data from the Swedish CArdioPulmonary bioImage Study (SCAPIS), the main purposes of this study were to (1) define age-dependent and population-specific reference ranges for echocardiographic diastolic function variables, and subsequently (2) characterise diastolic function and identify risk factors for diastolic dysfunction in individuals with pre-diabetes or diabetes.

## Methods

### Study population

SCAPIS is a population-based study including 30 154 men and women, aged 50–64 years, randomly recruited from the census register in six Swedish university cities. The multicentre core study protocol has been previously described.^18^ The 5058 subjects recruited during 2015–2018 in Linköping were additionally invited to undergo a TTE examination. The study has been ethically approved (Dnr. 2010/228-31M, 2018/278-31, 2022–02122-02), conforms with the principles outlined in the Declaration of Helsinki and participants gave written informed consent.

**Figure 1:**
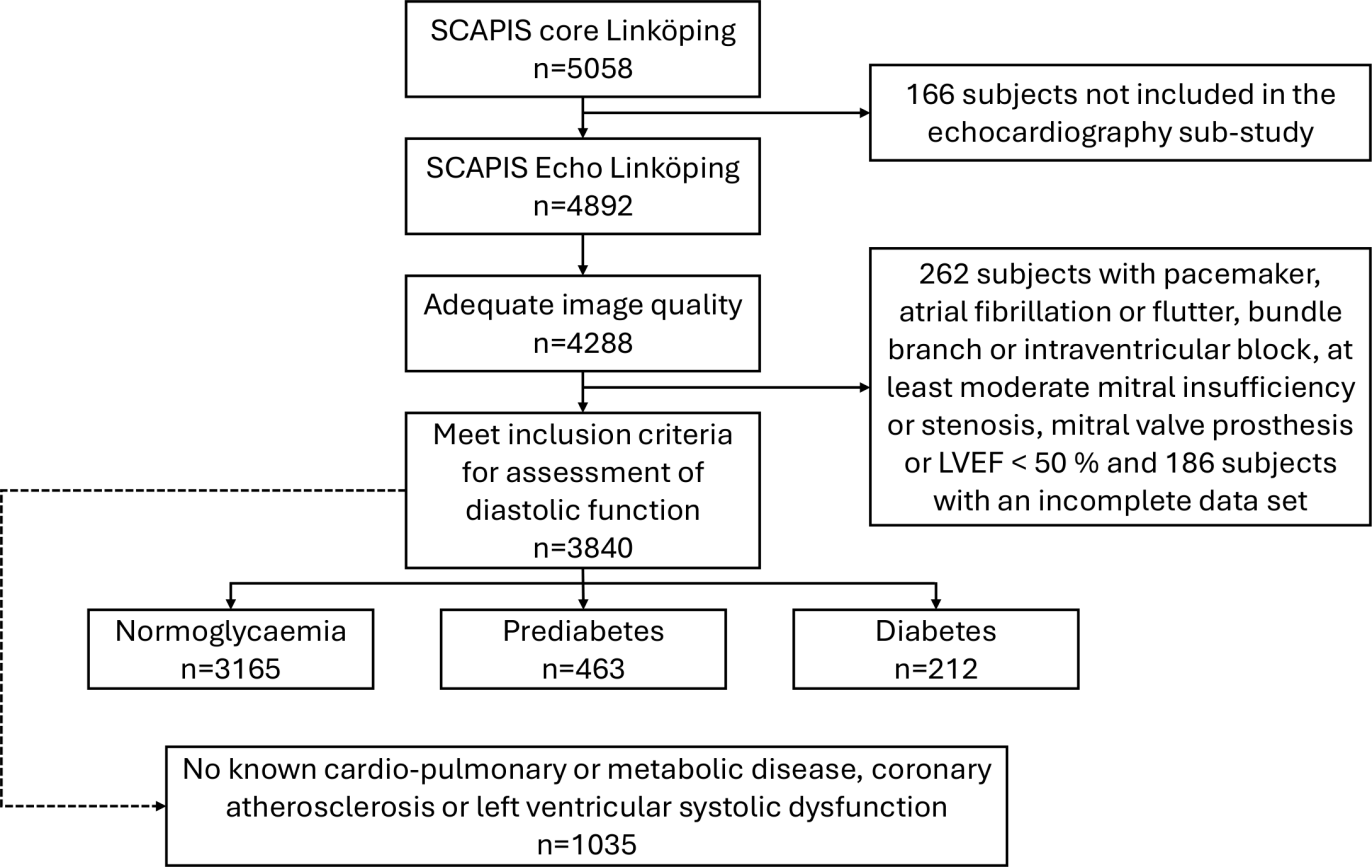
Flow chart depicting study inclusion. Echo, echocardiography; LVEF, left ventricular ejection fraction; SCAPIS, Swedish CArdioPulmonary bioImage Study.

Participants in the SCAPIS Linköping echocardiography study were eligible for inclusion. We excluded subjects with pacemaker treatment, atrial fibrillation/flutter, bundle branch or intraventricular block, at least moderate mitral insufficiency or stenosis or mitral valve prosthesis, which prevent a comprehensive evaluation of diastolic function. Subjects with reduced image quality and/or reduced LVEF <50% were also excluded (figure 1).

Medical history was retrieved from questionnaires. Dyspnoea on exertion was defined by a positive answer to the question ‘Are you troubled by shortness of breath when hurrying on level ground or walking up a slight hill?’ (adopted from the European Community Respiratory Health Survey Questionnaires^19^). A 12-lead ECG at study inclusion identified subjects with atrial fibrillation/flutter, bundle branch/intraventricular block or pacemaker.

Brachial arterial blood pressure was automatically measured (Omron M10-IT, Omron healthcare CO, Kyoto, Japan) twice in each arm after a 5-minute rest and mean measurements were used. Glucose, glycated haemoglobin (HbA1c), creatinine, high-sensitivity C-reactive protein (hsCRP) and N-terminal pro-B natriuretic peptide (NT-proBNP) were analysed after an overnight fast. Analyses were performed at the Department of Clinical Chemistry, Linköping University Hospital, which is accredited according to ISO-IEC 15189.

### CT

The cardiac CT in SCAPIS has been described in detail elsewhere.^18 20^ In short, coronary artery calcium was assessed in non-contrast enhanced images from a multisliced CT scanner (Siemens, Somatom Definition Flash, Siemens Healthineers, Erlangen, Germany). Coronary artery calcium was identified and scored in each of the main coronary arteries (left main/left anterior descending, circumflex and right coronary arteries) using the syngo.via calcium scoring software (Volume Wizard, Siemens Healthineers, Erlangen, Germany) and total Coronary Artery Calcium Score (CACS) was calculated according to Agatston.^21^ Coronary atherosclerosis according to the 18 coronary segment model defined by the Society of Cardiovascular CT^22^ was assessed by analysis of contrast-enhanced coronary CT angiography (CCTA) images. Total coronary artery atherosclerotic burden was calculated using the Segment Involvement Score (SIS).^23^

### Transthoracic echocardiography

A comprehensive TTE was performed using a GE Vivid E95 echocardiography system and M5Sc probes (GE Healthcare, Chicago, Illinois, USA). Measurements were made offline using EchoPAC V.201 (GE Healthcare). Chamber size and function were evaluated according to recommendations^24 25^ and valve pathology was graded according to guidelines.^26^

Left ventricular end-diastolic diameter and end-diastolic septal and posterior wall thickness were assessed from the parasternal long-axis view. Left ventricular mass was calculated according to Devereux.^27^ Mitral inflow velocities were measured by pulsed-wave Doppler at the mitral leaflets tips. Tissue-Doppler images of the apical four-chamber view were recorded and early diastolic velocity (e’) in the basal septum was measured offline using Q-analysis in EchoPAC. Mitral E to A-wave (E/A) and mitral E to septal e’ (E/e’) ratios were calculated. Left atrial end-systolic area was measured in the apical four-chamber view and left atrial end-systolic volume was estimated using Simpson’s uniplane method. Left ventricular end-diastolic volume and LVEF were measured using the semi-automated quantification tool Auto-EF in EchoPAC. Global longitudinal strain (GLS) was measured from the three apical views using the semiautomated Automated Functional Imaging in EchoPAC. Measures of chamber size and mass were indexed to body surface area by the Mosteller formula.^28^

### Classification of glycaemic status

Based on their fasting glucose status, HbA1c level and/or self-reported known diabetes the participants were classified as either having normoglycaemia (fasting plasma (fP)-glucose <6.1 mmol/L and HbA1c <42 mmol/mol), pre-diabetes (impaired fasting glucose with fP-glucose 6.1–6.9 mmol/L and/or elevated HbA1c in the range 42–47 mmol/L) or diabetes (fP-glucose ≥7.0 mmol/L and/or HbA1c ≥48 mmol/L and/or self-reported known diabetes), as previously described.^29^

### Statistics

Analyses were performed using SPSS Statistics V.27 (IBM, Armonk, New York, USA). Continuous variables are presented as mean±SD if normally distributed or median (IQR) if non-normally distributed. Categorical variables are presented as numbers (per cent). For group comparisons, independent samples t-test, Mann-Whitney U-test, one-way analysis of variance (ANOVA), Kruskal-Wallis one-way ANOVA or χ² test were used depending on type and distribution of data. Correlations were assessed using Pearson’s method.

For determination of reference values for diastolic function variables, subjects with a history of myocardial infarction, coronary artery bypass grafting (CABG), percutaneous coronary intervention (PCI), angina pectoris, heart failure, stroke, hypertension, chronic obstructive pulmonary disease, chronic bronchitis or emphysema and/or hyperlipidaemia were excluded, as were subjects with body mass index (BMI) >30 kg/m^2^, CACS >0, SIS >0 and/or GLS of −17% or lower (figure 1). Upper and lower limits of normal were determined by the 5th and 95th percentiles. Some diastolic function variables are age-dependent^30^ and age-dependency of diastolic function variables was assessed to create separate reference ranges based on age-tertiles (50–54, 55–59 and 60–64 years) when appropriate. Diastolic function indices were compared between glycaemic groups both as continuous variables and categorical variables (frequency of abnormal values). We additionally compared the number of abnormal diastolic function variables.

E/e’ was chosen as the main outcome variable primarily as higher E/e’ has previously been found to be a strong predictor of cardiovascular events in patients with diabetes.^31^ The relationship between E/e’ ratio above upper limit of normal (ULN) and potential determinants of diastolic dysfunction in pre-diabetes and diabetes (age, waist circumference, left ventricular mass index (LVMI), HbA1c and hsCRP as continuous variables, and sex, hypertension, hyperlipidaemia, CACS ≥400 and a diagnosis of ischaemic heart disease as defined by previous myocardial infarction, PCI, CABG or angina pectoris as categorical variables) were analysed by univariable and multivariable logistic regression. Variables with a p value <0.1 in univariable analysis were included in the multivariable analysis. Separate analyses were performed after (1) excluding subjects with a diagnosis of ischaemic heart disease and (2) for subjects without coronary atherosclerosis (excluding subjects with CACS >0 and/or SIS >0) and hypertension. For the latter analysis, systolic blood pressure was included in the logistic regression. We tested for multicollinearity by variance of inflation and tolerance, which were consistently well below the accepted thresholds for all variables included in multivariable analyses.

A p value <0.05 was considered statistically significant.

## Results

### Reference ranges for diastolic function variables

1035 (27%) of included subjects had no known cardiovascular (including hypertension), metabolic or pulmonary disease, no coronary atherosclerosis on coronary CT and CCTA, and normal left ventricular systolic function (figure 1). Data from their examinations were used to determine reference ranges for diastolic function variables. Age-dependency of diastolic function variables are presented in online supplemental tables 1-2 and reference ranges in online supplemental table 3.

### Diastolic dysfunction in pre-diabetes and diabetes

**Figure 2:**
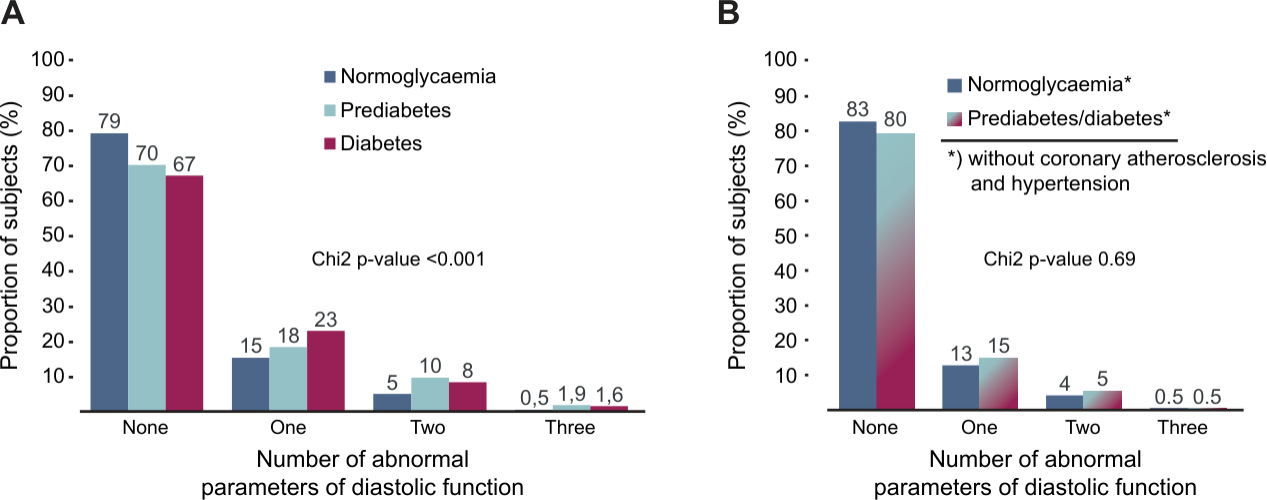
Number of abnormal diastolic function variables in normoglycaemia, pre-diabetes and diabetes.

In the full sample, 3165 (82%) were normoglycaemic, 463 (12%) had pre-diabetes and 212 (6%) had diabetes. Doppler variables of diastolic function were impaired in pre-diabetes and diabetes, compared with normoglycaemia (online supplemental table 4). For E/e’ and E/A ratios, impairments were worse in diabetes than pre-diabetes. Overall normal diastolic function variables were less frequent in pre-diabetes and diabetes, whereas multiple diastolic function variables out of normal range were more frequent (online supplemental table 4 and figure 2A).

Subjects with pre-diabetes or diabetes and elevated E/e’ (15% of the pre-diabetes/diabetes group) had higher blood pressure, higher hsCRP, were more frequently overweight, had more severe coronary atherosclerosis, more often hypertension, ischaemic heart disease, elevated NT-proBNP and dyspnoea on exertion. A comprehensive comparison is shown in table 1 and online supplemental table 5. In multivariable logistic regression, greater waist circumference and high CACS were associated with elevated E/e’ in pre-diabetes and diabetes (table 2). Results remained after excluding subjects with a diagnosis of ischaemic heart disease (online supplemental table 6).

**Table 1:** Characteristics of subjects with pre-diabetes/diabetes and E/e’ below or above upper limit of normal

	E/e’ below ULN n=545	E/e’ above ULN n=103	P value
Age, *years*	58.1 (54.8–61.8)	60.3 (54.8–63.2)	0.022
Female sex, *n* (*%*)	251 (46.1)	47 (45.6)	0.937
Body mass index, *kg/m^2^*	27.7±4.3	30.2±4.3	<0.001
Waist circumference, *cm*	100±12.1	101.9±12.2	<0.001
Systolic blood pressure, *mm Hg*	134.7±17	141.5±18	<0.001
Diastolic blood pressure, *mm Hg*	83.6±9.7	87.3±11.1	0.001
Ever-smokers, *n* (*%*)	251 (46.1)	47 (45.6)	0.991
Dyspnoea on exertion, *n (%*)	40 (7.3)	16 (15.5)	0.006
Cardiovascular disease			
Previous myocardial infarction, *n* (*%*)	10 (1.8)	7 (6.8)	0.003
Previous CABG/PCI, *n* (*%*)	8 (1.5)	8 (7.8)	<0.001
Angina pectoris, *n* (*%*)	5 (0.9)	2 (1.9)	0.344
Hypertension, *n* (*%*)	158 (29)	49 (47.6)	<0.001
Hyperlipidaemia, *n (%*)	106 (19.4)	26 (25.2)	0.150
Previous stroke, *n* (*%*)	7 (1.3)	4 (3.9)	0.057
Biochemistry			
HbA1c, *mmol/mol*	39 (35–43)	40 (36–45)	0.084
eGFR, *ml/min*	98.3 (83.4–114.8)	104.1 (89.5–115.5)	0.037
hsCRP, *mg/L*	1.1 (0.6–2.6)	1.2 (0.7–3.2)	0.046
NT-proBNP, *pg/L*	45.7 (26–77.5)	51 (28.7–95.9)	0.132
NT-proBNP ≥125 pg/L	42 (7.9)	19 (18.4)	0.001
Coronary atherosclerosis			
CACS 0, *n* (*%*)	261 (47.9)	36 (35)	<0.001
CACS 1–99, *n* (*%*)	182 (33.4)	33 (32)	
CACS 100–399, *n* (*%*)	59 (10.8)	12 (11.7)	
CACS ≥400, *n* (*%*)	23 (4.2)	17 (16.5)	
SIS ≥4, *n* (*%*)	63 (11.6)	20 (19.4)	0.017

E/e’ was available for 648 (96%) of 675 subjects with pre-diabetes/diabetes. Values are mean±SD, median (IQR) or number (per cent).

CABG, coronary artery bypass grafting; CACS, Coronary Artery Calcium Score; E/e’, mitral E to septal e’ ratio; eGFR, estimated glomerular filtration rate; HbA1c, glycated haemoglobin; hsCRP, high-sensitivity C-reactive protein; NT-proBNP, N-terminal pro-B natriuretic peptide; PCI, percutaneous coronary intervention; SIS, Segment Involvement Score; ULN, upper limit of normal.

**Table 2:** Univariable and multivariable logistic regression of relationship between elevated E/e’ and potential determinants of diastolic dysfunction in pre-diabetes/diabetes

Variables	Univariable models		Multivariable model	
	OR (95% CI)	P value	OR (95 % CI)	P value
Age	1.059 (1.007 to 1.113)	0.024	1.029 (0.974 to 1.087)	0.311
Female sex	0.983 (0.644 to 1.500)	0.937	–	–
Waist circumference	1.041 (1.023 to 1.060)	<0.001	1.034 (1.013 to 1.054)	<0.001
Hypertension	2.223 (1.448 to 3.412)	<0.001	1.557 (0.962 to 2.518)	0.071
Hyperlipidaemia	1.398 (0.855 to 2.289)	0.182	–	–
Left ventricular mass index	1.009 (0.977 to 1.021)	0.129	–	–
HbA1c	1.016 (1.000 to 1.032)	0.055	1.001 (0.982 to 1.019)	0.952
hsCRP	1.011 (0.980 to 1.042)	0.493	–	–
CACS ≥400	4.581 (2.345 to 8.946)	<0.001	2.897 (1.373 to 6.113)	0.005
Ischaemic heart disease*	4.225 (1.881 to 9.486)	<0.001	2.696 (0.831 to 8.748)	0.099

Variables with a p value <0.1 in univariable analysis were included in the multivariable analysis.

* Defined as previous myocardial infarction, percutaneous coronary intervention, coronary artery bypass grafting or angina pectoris.

CACS, coronary artery calcium score; E/e’, mitral E to septal e’ ratio; HbA1c, glycated haemoglobin; hsCRP, high-sensitivity C-reactive protein.

19% of subjects with diabetes, and 38% of subjects with pre-diabetes were free from coronary atherosclerosis and hypertension. Subjects with pre-diabetes or diabetes without coronary atherosclerosis and hypertension still had a discrete impairment of diastolic function variables and more frequently E/e’ above ULN, compared with subjects with normoglycaemia without coronary atherosclerosis and hypertension (online supplemental table 4). The frequency of diastolic function variables outside of normal range did however not differ between normoglycaemia and pre-diabetes/diabetes without coronary atherosclerosis and hypertension (figure 2B). In multivariable logistic regression, systolic blood pressure was the only factor associated with elevated E/e’ in pre-diabetes and diabetes without coronary atherosclerosis and hypertension (online supplemental table 7).

## Discussion

Our data show that individuals with pre-diabetes or diabetes have an increased prevalence of diastolic dysfunction. The frequency of abnormal diastolic function variables was similar between the two conditions, although with a somewhat greater impairment for diabetes than pre-diabetes. Our comprehensive analysis shows that diastolic dysfunction was associated with traditional cardiovascular risk factors, particularly hypertension, central adiposity and coronary atherosclerosis. Higher blood pressure was still the only factor associated with diastolic dysfunction among subjects free from coronary atherosclerosis and hypertension.

Given the complexity and age-dependency of echocardiographic variables used to assess diastolic function, in combination with measurement errors, methodological differences in measurements and changing diagnostic algorithms,^32^ it is a challenge to determine the prevalence of diastolic dysfunction in the population in a standardised and reproducible manner. We used a subset of the study population with no known cardiopulmonary or metabolic disease, no coronary atherosclerosis and normal left ventricular systolic function to determine population-specific reference values for diastolic function variables, including septal e’, E/e’ ratio, E/A ratio and left atrial volume index. Despite the narrow age-span of the study participants, we found that diastolic Doppler parameters varied with age and specific reference values were retrieved per age tertile (ie, 50–54, 55–59 and 60–64 years). We believe this approach reduces the risk of age-related misclassification of diastolic dysfunction in the present study. In multivariable analyses, we additionally adjusted for age to account for any residual age-related effects not fully captured by the adapted reference ranges.

As compared with subjects with normoglycaemia, the number of abnormal diastolic function variables was higher in pre-diabetes and diabetes. Subjects with pre-diabetes or diabetes were almost twice as likely to have two abnormal diastolic function variables and almost four times as likely to have three abnormal diastolic function variables as subjects with normoglycaemia. Nevertheless, only approximately 10–12% of subjects with pre-diabetes or diabetes had two and only 1.6–1.9% had three abnormal diastolic function variables. Previous general population-based studies report the prevalence of diastolic dysfunction in diabetes to be 23–54%, but all defined diastolic dysfunction differently.^1333^,^37^ When there was a normoglycaemic reference group, the prevalence of diastolic dysfunction was, however, 1.4–1.8 times higher for subjects with diabetes irrespective of the different definitions^36 37^ and these numbers appear to be comparable also to our results. In the Olmsted County study, diastolic dysfunction was defined as E/e’ >15, which rendered a prevalence of diastolic dysfunction of 23% in diabetes.^34^ This is slightly higher than our frequencies of 15 and 18% for elevated E/e’ in subjects with pre-diabetes or diabetes, respectively. Whereas Pareek *et al*,^36^ in line with our findings, reported elevated E/e’ ≥13 in 15% of subjects with diabetes. A high E/e’ was however not more common among subjects with pre-diabetes in their study. Besides differences in the definition of diastolic dysfunction, population-related factors may obviously also contribute to the observed discrepancies. Our findings of a higher frequency of dyspnoea on exertion and elevated NT-proBNP levels in subjects with elevated E/e’ suggest that at least 15–20% of those with high E/e’, corresponding to 2–3% of the entire population with pre-diabetes or diabetes, may indeed have HFpEF. This number is much lower than that reported by Boonman-de Winter *et al*^13^ who, by echocardiography and a clinical assessment, found that HFpEF was present in 23% of a diabetes cohort from the general population. The mean age was however higher and cardiovascular comorbidities were more common in their cohort.

As E/e’ has been found to be an independent predictor of heart failure^34^ and other major cardiac events,^31^ we used E/e’ above ULN as our primary outcome variable for diastolic dysfunction. The most striking differences between subjects with pre-diabetes or diabetes with or without elevated E/e’ were the higher prevalence of hypertension (48 vs 29%) and the more severe degree of coronary atherosclerosis on coronary CT and CCTA in the group with elevated E/e’. In multivariable logistic regression, only waist circumference and high CACS were however significantly associated with elevated E/e’. We chose waist circumference rather than BMI as our adiposity metric as it more directly reflects the metabolically active visceral fat depots and has been shown to better capture obesity-related cardiometabolic risk.^38^ In line with our findings, Packer *et al* recently proposed that central adiposity, acting through neurohormonal activation and adipokine imbalance, is generally a more important risk factor for HFpEF than hypertension today.^8^ No previous study has to the best of our knowledge, analysed CACS and coronary atherosclerosis by CT angiography in a large, population-based cohort of subjects with pre-diabetes and diabetes with or without signs of diastolic dysfunction. Our findings show that coronary atherosclerosis with high CACS appears to be an important risk factor for diastolic dysfunction also in the absence of a clinical diagnosis of ischaemic heart disease.

In a previous study on the entire SCAPIS cohort of approximately 30 000 subjects, coronary atherosclerosis was more common in subjects with pre-diabetes or diabetes than in normoglycaemia^29^ and subjects in SCAPIS with CACS ≥400 all had coronary atherosclerosis with approximately 45% showing stenosis of 50%.^20^ Interestingly, Aljizeeri *et al*^39^ revealed that impairment of the coronary microcirculation, and/or diffuse non-obstructive coronary artery disease, with subsequent reduction of coronary blood flow appears to be common in diabetes. It seems plausible that, beside obstructive coronary artery disease, such impairment may also contribute to the relationship between CACS and diastolic dysfunction observed in our population.^29^ In accordance, coronary atherosclerotic burden in the SCAPIS population is indeed related to reduced microvascular function.^40^

38% of subjects with pre-diabetes and 19% of subjects with diabetes in this study were free from coronary atherosclerosis and hypertension. When analysing this subset, subjects with pre-diabetes or diabetes still had slightly impaired diastolic function variables and more frequently elevated E/e’, as compared with subjects with normoglycaemia without coronary atherosclerosis and hypertension. Although those with a clinical diagnosis of hypertension were excluded in this subgroup analysis, systolic blood pressure was interestingly the only variable associated with diastolic dysfunction in multivariable logistic regression. Although our results do not exclude the concept of a diabetes-related cardiomyopathy, they challenge the concept of a clinically relevant, isolated diabetic cardiomyopathy characterised by reduced left ventricular function in the absence of hypertension and coronary artery disease.

### Limitations

Our database lacks data on tricuspid regurgitation velocity, which could therefore not be included in the evaluation of diastolic function. Our experience is, nevertheless, that a well-defined and reliable tricuspid regurgitation signal is infrequently recorded in a general population sample. Importantly, we did in accordance with previous studies,^34 36^ use E/e’, which is strongly related to cardiovascular risk,^31 34^ as our primary outcome variable for diastolic dysfunction. Of note, measures of septal e’ were not made directly by pulsed wave tissue Doppler, but offline from colour tissue Doppler recordings, which are known to render slightly lower values, although with excellent agreement to pulse wave Doppler registrations.^41^ This does not impact the conclusions of the present study but needs to be taken into account for comparisons with other studies and if the derived reference values were to be applied in another cohort. Additionally, since HFpEF is a clinical diagnosis, we could not determine its ‘true’ frequency in the population. Pre-diabetes was defined merely by impaired fasting glucose and/or elevated HbA1c as we did not have data from an oral glucose tolerance test. The study includes subjects within the age-span 50–64 years, which affects the generalisability of the results. This is nevertheless an age-span where cardiovascular diseases begin to manifest and when there is still time for preventive actions. Finally and importantly, this is a cross-sectional cohort study, which should be considered exploratory and no definitive conclusions may be drawn as to causality. We also recognise that the pathophysiology of diastolic dysfunction is multifaceted with a complex interplay between metabolic and neurohormonal factors, the vascular system and haemodynamic burden that affect both cardiac structure and function. The causal pathways are not always clear and we acknowledge that there may be potential overadjustment in our models. The ongoing longitudinal follow-up of the SCAPIS cohort may help to determine patterns of progression.

## Conclusions

In this population-based cohort, diastolic dysfunction was as common in pre-diabetes as in established diabetes, suggesting that myocardial functional changes are present already at early stages of dysglycaemia. Diastolic dysfunction was mainly associated with central adiposity, hypertension and subclinical coronary atherosclerosis, even in the absence of clinically overt ischaemic heart disease. Among individuals without diagnosed hypertension or coronary atherosclerosis, systolic blood pressure was still the only independent predictor.

These findings indicate that diastolic dysfunction in dysglycaemia is largely linked to concomitant modifiable cardiometabolic risk factors rather than an isolated diabetic cardiomyopathy. The results emphasise the importance of early risk factor identification and management, including weight control and blood pressure optimisation, to reduce the future burden of HFpEF.

## Supplementary material

10.1136/openhrt-2026-004052 online supplemental table 1

## Data Availability

Data are available upon reasonable request.
