# Supplementary material for: Diastolic dysfunction is equally common in pre-diabetes and diabetes and associated with concomitant cardiometabolic risk factors
Source: Open Heart. 2026 May 20;13(1):e004052. doi: 10.1136/openhrt-2026-004052 (PMC13202158; doi:10.1136/openhrt-2026-004052)
Supplement: online supplemental table 1 [file openhrt-13-1-s001.docx]

**Supplemental tables**

**Supplemental table 1. Correlation between diastolic function variables and age in subjects with no known cardiovascular, metabolic or pulmonary disease, nor signs of coronary atherosclerosis on CCTA and normal left ventricular deformation**

|  | **Pearson’s correlation coefficient** | **P value** |
| --- | --- | --- |
| Septal e’, *cm/s* | -0.352 | <0.001 |
| E/e’ ratio, *unitless* | 0.172 | <0.001 |
| E/A ratio, *unitless* | -0.332 | <0.001 |
| LAVI_,_ *ml/m^2^* | -0.008 | 0.798 |

Abbreviations: septal e’, early diastolic myocardial velocity at the septal mitral annulus; E/e’ ratio, ratio between early diastolic transmitral flow and mitral annular motion velocities; E/A ratio, ratio between early and late diastolic transmitral flow velocities; LAVI, end-systolic left atrial volume index.

**Supplemental table 2. Comparison of diastolic function variables between three age groups in subjects with no known cardiovascular, metabolic or pulmonary disease, nor signs of coronary atherosclerosis on CCTA and normal left ventricular deformation**

|  | **Age 50-54 years**  **n=470** | **Age 55-59 years**  **n=324** | **Age 60-64 years**  **n=241** | **P value** |
| --- | --- | --- | --- | --- |
| Septal e’, *cm/s* | 8.0±1.6* | 7.2±1.5* | 6.7±1.5* | <0.001 |
| E/e’ ratio, *unitless* | 8.7 (7.1-10.5) | 9.1 (7.4-10.9) | 10.0 (8.3-11.9) † | <0.001 |
| E/A ratio, *unitless* | 1.4 (1.2-1.7)* | 1.3 (1.0-1.5)* | 1.1 (0.9-1.3)* | <0.001 |
| LAVI_,_ *ml/m^2^* | 26.2±8.0 | 24.9 ±7.3 | 26.1±8.9 | 0.057 |

Values are mean±standard deviation or median (interquartile range) depending on the distribution of data. For *post hoc* pairwise comparisons, a * indicates a statistically significant difference, as compared to the two other age groups, a † indicates a statistically significant difference as compared to 50-54 and 55-59 year old. Abbreviations: septal e’, early diastolic myocardial velocity at the septal mitral annulus; E/e’ ratio, ratio between early diastolic transmitral flow and mitral annular motion velocities; E/A ratio, ratio between early and late diastolic transmitral flow velocities; LAVI, end-systolic left atrial volume index.

**Supplemental table 3. Reference values for diastolic function variables**

|  | **Age 50-54 years**  **n=470** | | | **Age 55-59 years**  **n=324** | | | **Age 60-64 years**  **n=241** | | |
| --- | --- | --- | --- | --- | --- | --- | --- | --- | --- |
|  | Median | 5^th^ percentile | 95^th^ percentile | Median | 5^th^ percentile | 95^th^ percentile | Median | 5^th^ percentile | 95^th^ percentile |
| Septal e’, *cm/s* | 8.0 | 5.7 | 10.7 | 7.2 | 4.6 | 9.7 | 6.6 | 4.3 | 9.6 |
| E/e’ ratio, *unitless* | 8.7 | 5.9 | 13.6 | 9.1 | 5.8 | 14.3 | 10.0 | 6.3 | 14.5 |
| E/A ratio, *unitless* | 1.4 | 0.9 | 2.3 | 1.3 | 0.8 | 2.0 | 1.1 | 0.7 | 1.9 |
|  | **All**  **n=1035** | | | | | | | | |
|  | Median | | | 5^th^ percentile | | | 95^th^ percentile | | |
| LAVI_,_ *ml/m^2^* | 24.8 | | | 14.4 | | | 40.8 | | |

Abbreviations: septal e’, early diastolic myocardial velocity at the septal mitral annulus; E/e’ ratio, ratio between early diastolic transmitral flow and mitral annular motion velocities; E/A ratio, ratio between early and late diastolic transmitral flow velocities; LAVI, end-systolic left atrial volume index. There was no relationship between age and LAVI.

**Supplemental table 4. Diastolic function variables in subjects with normoglycaemia, prediabetes or diabetes**

|  | **Normoglycaemia**  **n=3165** | **Prediabetes**  **n=463** | **Diabetes**  **n=212** | **P value^1^** | **Normoglycaemia without coronary atherosclerosis and hypertension**  **n=1660** | **Prediabetes or diabetes without coronary atherosclerosis and hypertension**  **n=217** | **P value^2^** |
| --- | --- | --- | --- | --- | --- | --- | --- |
| Septal e’, *cm/s* | 6.9±1.7* | 6.5±1.7† | 6.2±1.5† | <0.001 | 7.2±1.7 | 6.8±1.5 | 0.003 |
| E/e’, *unitless* | 9.4 (7.7-11.6)* | 10.2 (8.3-12.6)* | 11.2 (9.0-13.5)* | <0.001 | 9.2 (7.5-11.1) | 9.5 (7.9-12.2) | 0.017 |
| E/A ratio, *unitless* | 1.2 (1.0-1.5)* | 1.1 (0.9-1.4)* | 1.0 (0.9-1.2)* | <0.001 | 1.2 (1.0-1.5) | 1.2 (1.0-1.4) | 0.020 |
| LAVI_,_ *ml/m^2^* | 25.6±8.2 | 25.7±7.6 | 25.2±8.6 | 0.765 | 25.7±8.4 | 25.4±7.7 | 0.463 |
| Septal e’ below LLN | 307 (9.9) | 72 (16) | 23 (11.4) | <0.001 | 135 (8.3) | 19 (9.0) | 0.791 |
| E/e’ above ULN | 217 (7.1) | 67 (15) | 36 (18) | <0.001 | 88 (5.5) | 21 (10) | 0.014 |
| E/A ratio below ULN | 186 (5.9) | 41 (8.9) | 28 (13.2) | <0.001 | 88 (5.3) | 12 (5.9) | 0.872 |
| LAVI above ULN | 154 (5) | 15 (3.4) | 11 (5.4) | 0.289 | 68 (4.2) | 7 (3.4) | 0.589 |

Values are mean±standard deviation or median (interquartile range) depending on the distribution of data. ^1^P value for comparison between the normoglycaemia, prediabetes, and diabetes groups. For *post hoc* pairwise comparisons, a * indicates a statistically significant difference, as compared to the two other groups, a † indicates a statistically significant difference only as compared to normoglycaemia. ^2^P value for comparison between normoglycaemia without coronary atherosclerosis and hypertensions and prediabetes or diabetes without coronary atherosclerosis and hypertension. Abbreviations: septal e’, early diastolic myocardial velocity at the septal mitral annulus; E/e’ ratio, ratio between early diastolic transmitral flow and mitral annular motion velocities; E/A ratio, ratio between early and late diastolic transmitral flow velocities; LAVI, end-systolic left atrial volume index.

**Supplemental table 5. Echocardiographic characteristics of subjects with prediabetes/diabetes and E/e’ below or above upper limit of normal**

|  | **E/e’ below ULN**  **n=545** | **E/e’ above ULN**  **n=103** | **P value** |
| --- | --- | --- | --- |
| LVEDDI, *mm/m^2^* | 25±2.6 | 24.5±4.6 | 0.076 |
| LVEDVI, *ml/m^2^* | 50.4±10.6 | 46.2±10.7 | <0.001 |
| LVMI, *g/m^2^* | 76.9±19.6 | 80.7±21.9 | 0.128 |
| LVEF, *%* | 59.5±4.5 | 58.8±4.3 | 0.185 |
| GLS, *%* | -19.8±2.1 | -19.8±2.1 | 0.985 |
| TAPSE, *mm* | 24.7±3.8 | 24.1±3.8 | 0.120 |
| RVFW s’, *cm/s* | 10.8±1.9 | 10.2±2.3 | 0.014 |
| Septal e’, *cm/s* | 6.8±1.5 | 4.7±1.1 | <0.001 |
| E/e’, *unitless* | 9.9 (8.3-11.7) | 16.5 (15.1-18.6) | <0.001 |
| E/A ratio, *unitless* | 1.1 (0.9-1.4) | 1.0 (0.9-1.2) | 0.044 |
| LAVI, *ml/m^2^* | 25.5±7.8 | 26.3±7.8 | 0.352 |

Meaurements of E/e’ was available for 648 (96%) of the 675 subjects with prediabetes or diabetes. Vales are mean±standard deviation, median (interquartile range) or absolute number (percent). Abbreviations: E/A ratio, ratio between early and late diastolic transmitral flow velocities; E/e’ ratio, ratio between early diastolic transmitral flow and mitral annular motion velocities; GLS, global longitudinal strain; LAVI, end-systolic left atrial volume index; LVEDDI, left ventricular end-diastolic diameter index; LVEDVI, left ventricular end-diastolic volume index; LVEF, left ventricular ejection fraction; RVFW s’, systolic myocardial velocity at the free wall tricuspid annulus; septal e’, early diastolic myocardial velocity at the septal mitral annulus; TAPSE, tricuspid annular plane systolic excursion; ULN, upper limit of normal.

**Supplemental table 6. Uni- and multivariable logistic regression describing the relationship between elevated E/e’ and potential determinants of diastolic dysfunction in subjects with prediabetes or diabetes without a clinical diagnosis of ischaemic heart disease***

| **Variables** | **Univariable models** | | **Multivariable model** | |
| --- | --- | --- | --- | --- |
|  | **OR (95% CI)** | **P value** | **OR (95 % CI)** | **P value** |
| Age | 1.039 (0.986-1.094)) | 0.153 | - | - |
| Female sex | 1.034 (0.664-1.611) | 0.881 | - | - |
| Waist circumference | 1.044 (1.024-1.063) | <0.001 | 1.032 (1.008-1.057) | 0.008 |
| Hypertension | 2.128 (1.355-3.343) | 0.001 | 1.416 (0.792-2.532) | 0.240 |
| Hyperlipidaemia | 1.320 (0.774-2.253) | 0.308 | - | - |
| LVMI | 1.012 (0.999-1.024) | 0.070 | 1.008 (0.995-1.021) | 0.243 |
| HbA1c | 1.022 (1.004-1.040) | 0.016 | 1.008 (0.984-1.032) | 0.508 |
| hsCRP | 1.013 (0.983-1.044) | 0.405 | - | - |
| CACS ≥ 400 | 5.051 (2.413-10.570) | <0.001 | 3.891 (1.533-9.872) | 0.004 |

Variables with a P value < 0.1 in univariate analysis were included in the multivariate analysis. Abbreviations: CACS, coronary artery calcium score; HbA1c, glycated haemoglobin; hsCRP, high-sensitivity C-reactive protein; LVMI, left ventricular mass index. *Defined as previous myocardial infarction, percutaneous coronary intervention, coronary artery by-pass grafting or angina pectoris.

**Supplemental table 7. Uni- and multivariable logistic regression of relationship between elevated E/e’ and potential determinants of diastolic dysfunction in subjects with prediabetes or diabetes without coronary atherosclerosis and hypertension**

| **Variables** | **Univariable models** | | **Multivariable model** | |
| --- | --- | --- | --- | --- |
|  | **OR (95% CI)** | **P value** | **OR (95 % CI)** | **P value** |
| Age | 1.016 (0.915-1.128) | 0.764 | - | - |
| Female sex | 2.087 (0.776-5.613) | 0.145 | - | - |
| Waist circumference | 1.012 (0.972-1.053) | 0.571 | - | - |
| Systolic blood pressure | 1.026 (1.001-1.053) | 0.042 | 1.027 (1.002-1.053) | 0.037 |
| Hyperlipidaemia | 0.759 (0.093-6.161) | 0.796 | - | - |
| LVMI | 1.001 (0.972-1.031) | 0.944 | - | - |
| HbA1c | 1.034 (0.995-1.075) | 0.088 | 1.038 (0.997-1.079) | 0.067 |
| hsCRP | 1.008 (0.936-1.086) | 0.833 | - | - |

Variables with a P value < 0.1 in univariate analysis were included in the multivariate analysis. Abbreviations: CACS, coronary artery calcium score; HbA1c, glycated haemoglobin; hsCRP, high-sensitivity C-reactive protein; LVMI, left ventricular mass index.
